# NEAT1 modulates neutrophil functions via glycolysis to restrain mucosal inflammation in ulcerative colitis

**DOI:** 10.3389/fimmu.2026.1802273

**Published:** 2026-08-19

**Authors:** Fengqin Zhu, Guiyuan Jin, Lihao Shi, Fengxian Dai, Cuimei Ma, Zongjing Hu, Yonghong Yang, Guanjun Dong, Guangxi Zhou

**Affiliations:** 1 Department of Gastroenterology, Affiliated Hospital of Jining Medical University, Jining Medical University, Jining, Shandong, China; 2 Medical Research Center, Affiliated Hospital of Jining Medical University, Jining Medical University, Jining, Shandong, China; 3 Institute of Immunology and Molecular Medicine, Jining Medical University, Jining, Shandong, China

**Keywords:** glycolysis, IBD - inflammatory bowel disease, NEAT1, neutrophils, ulcerative colitis

## Abstract

Neutrophils are essential for maintaining intestinal mucosal balance during ulcerative colitis (UC). The long noncoding RNA known as nuclear paraspeckle assembly transcript 1 (NEAT1) is associated with various inflammatory disorders. Nonetheless, the role of NEAT1 in influencing neutrophil immune responses in the context of UC remains unclear. In this study, we observed that NEAT1 expression was elevated in the inflamed mucosa of UC patients, showing a positive correlation with the disease activity. NEAT1 gene knockout (NEAT1 KO) mice exhibited less severe intestinal mucosal inflammation after dextran sulfate sodium (DSS) treatment. NEAT1 was predominantly expressed in neutrophils and elevated in neutrophils from UC patients. A deficiency in NEAT1 resulted in immune function remodeling of neutrophils, including a reduction in the production of pro-inflammatory cytokines, chemokines, reactive oxygen species, and neutrophil extracellular traps both *in vitro* and *in vivo*. Mechanistically, NEAT1 regulated neutrophil functions partially via glycolysis. Our research reveals a new mechanism through which NEAT1 influences the pathological development of UC by limiting the overactivation of neutrophils via glycolysis, providing a rationale for targeting NEAT1 in UC treatment.

## Graphical Abstract

**Figure:**
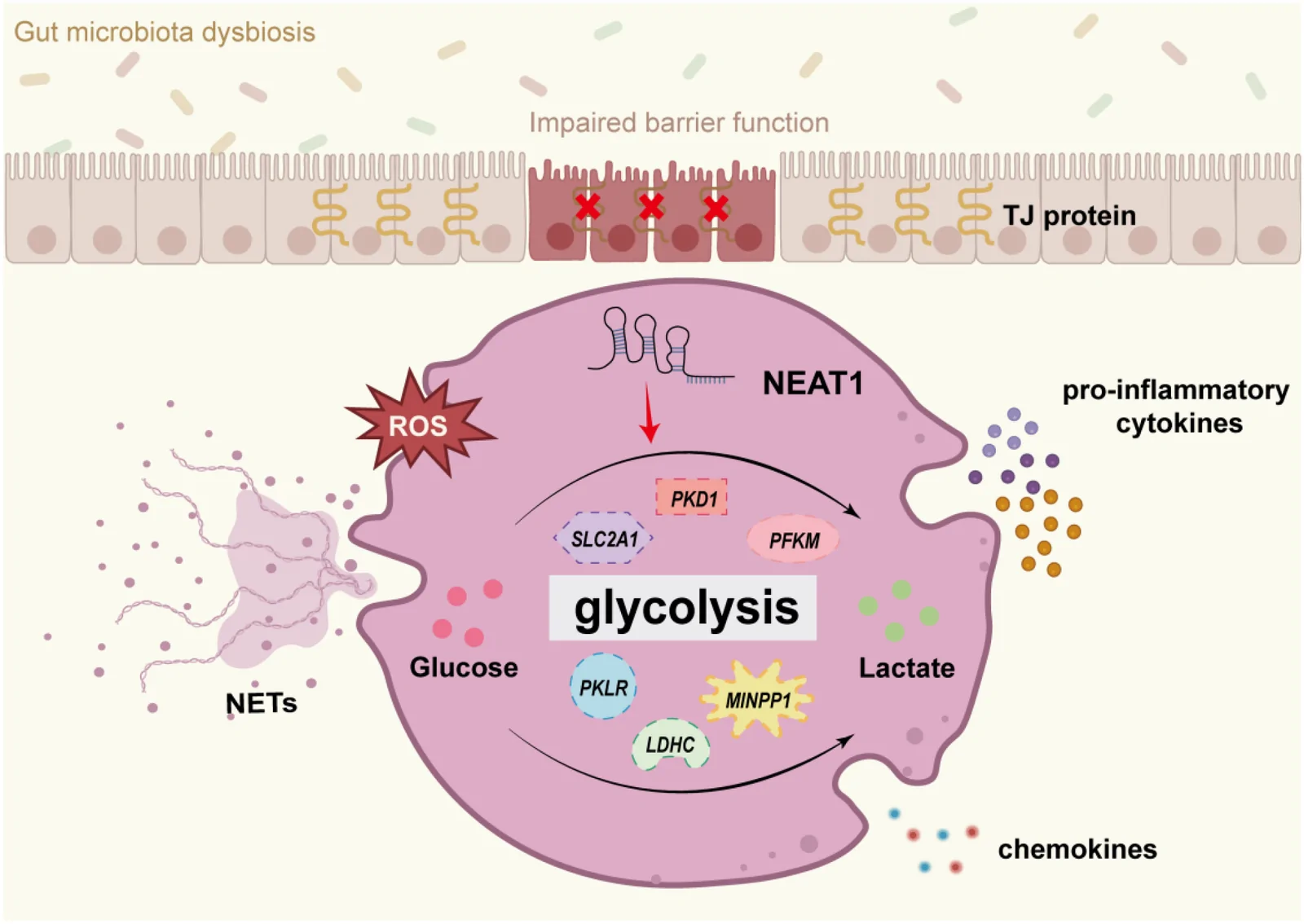
This research offers valuable perspectives on how NEAT1 affects the functionality of neutrophils during UC. NEAT1 downregulation leads to elevated neutrophil glycolysis, reduced ROS, NETs and pro-inflammatory factors, and ultimately relief of intestinal mucosal inflammation.

## 1 Introduction

Ulcerative colitis (UC) is an idiopathic and relapsing inflammatory disorder that is clinically marked by symptoms like abdominal discomfort, bloody diarrhea, and weight loss (1). It can affect individuals of any age, and shows a rising incidence alongside high morbidity and mortality rates globally (2). Present therapeutic strategies primarily aim to inhibit the aberrant immune response and inflammation in the gut. Despite recent advancements in the treatment of UC, instances of treatment failure are frequent (3, 4). Therefore, it is essential to clarify the immunological mechanisms underlying UC, which could pave the way for innovative precision medicine approaches in the future.

While the exact causes and mechanisms are still unclear, UC is widely thought to arise from dysfunction in the innate and/or adaptive immune responses within the intestinal mucosa, influenced by a mix of genetic, environmental, and microbial factors (5). Compromised innate immunity results in an inability to regulate altered intestinal microbiota, triggering the activation of the adaptive immune system and prompting a secondary inflammatory response that leads to tissue damage (6, 7). Neutrophils play a crucial role in the innate immune response in UC, and many studies indicate their involvement in the disease’s pathogenesis and progression (8). The excessive infiltration of activated neutrophils, along with the recruitment of additional active immune cells (such as macrophages, dendritic cells, T cells, and B cells), is linked to aggravated intestinal inflammation during the initial stages of UC (9). Nevertheless, these gathered neutrophils also have the capacity to degrade and eliminate pathogens through processes like phagocytosis, degranulation, release of reactive oxygen species (ROS), and the presence of neutrophil extracellular traps (NETs) (10, 11). Additionally, neutrophils facilitate angiogenesis and the proliferation of intestinal epithelial cells, contributing to the repair of the intestinal mucosal wounds (12, 13). Therefore, targeting the varied functions of neutrophils holds significant therapeutic promise.

Long noncoding RNAs (lncRNAs) constitute a category of RNA molecules that are greater than 200 nucleotides in length (14). While lncRNAs do not encode proteins, they are crucial to numerous biological functions and the development of diseases (15). Abnormal regulation of lncRNAs has been linked to processes such as tumor development, metastasis, and resistance to drugs (16, 17). Recent researches have revealed several lncRNAs that play roles in both innate and adaptive immunity. One such lncRNA, Nuclear Enriched Abundant Transcript 1 (NEAT1), exists in two forms: NEAT1_1 (3.7 kb) and NEAT1_2 (23 kb), and exhibits high expression levels in various immune cell types (18). NEAT1 has been shown to be involved in the progression of numerous inflammatory and immune-related diseases, including inflammatory bowel disease (IBD) (19–22). Several studies have demonstrated that NEAT1 is upregulated in intestinal tissues of UC patients and contributes to epithelial barrier dysfunction, cytokine regulation, metabolic changes, and NFκB-associated inflammatory signaling (23–28). Nevertheless, the role of NEAT1 in neutrophils, a key mediator of UC, has remained entirely unaddressed.

In this study, we determined the effects of NEAT1 on neutrophil functions in the context of UC for the first time. We found that NEAT1 was overexpressed in the intestinal mucosa of patients with UC and positively correlated with disease activity. NEAT1 gene knockout markedly alleviated DSS-induced colitis in mice, including reduced inflammatory cells infiltration, enhanced recovery of tight junction proteins, and improves intestinal microbiota dysbiosis. Notably, NEAT1 was highly expressed on neutrophils and elevated on neutrophils of UC patients. NEAT1 deficiency decreased the production and release of pro-inflammatory cytokines, chemokines, ROS, as well as NETs formation by neutrophils both *in vitro and in vivo*. In addition, we found that the modulation of NEAT1 on neutrophil immune-responses was mediated by glycolysis.

## 2 Materials and methods

### 2.1 Patients

Individuals diagnosed with UC and healthy control participants (HCs) were recruited from the Department of Gastroenterology at the Affiliated Hospital of Jining Medical University in Jining, Shandong, China, between May 2020 and September 2024. Diagnosis of UC was based on criteria that are standard for clinical evaluations, radiological assessments, endoscopic findings, and histological examinations. To assess the severity of the disease, we utilized recognized metrics, including the Mayo score, the ulcerative colitis endoscopic severity index (UCEIS), and the Dublin index for evaluating the extent of luminal inflammation in ulcerative colitis (29). Clinical indicators related to UC, such as C-reactive protein (CRP), hemoglobin (Hb), platelet (Plt) counts, and lymphocyte ratio, were collected. The characteristics of both the UC patients and the healthy controls participating in this research are outlined in **Table 1**. Approval for this study was granted by the Institutional Review Board for Clinical Research at the Affiliated Hospital of Jining Medical University, and all participants provided written informed consent before taking part in the study.

**Table 1:** The characteristics of UC patients and healthy donors in *in vitro* experiment.

Characteristics	Blood samples		*P*	Biopsy samples		*P*
	HC	UC		HC	UC	
Number of patients	19	21		40	39	
Age (y)	32.0 ± 4.7	40.5 ± 6.5	*P* < 0.001	36.2 ± 4.3	42.5 ± 8.8	*P* < 0.001
Gender			*P* = 0.775			*P* = 0.200
Male	10	12		21	26	
Female	9	9		19	13	
Disease duration (month)		60.5 ± 19.8			97.0 ± 41.7	*P* < 0.001
Disease extent (UC)						*P* = 0.915
E1		5			8	
E2		7			15	
E3		9			16	
Mayo Score						*P* = 0.934
Mild		5			10	
Moderate		8			16	
Severe		8			13	
CRP (mg/L)					33.16 ± 44.35	
Hemoglobin (g/L)					125.51± 15.63	
platelet (×10^9^)					350.6± 155.23	
Lymphocyte (×10^9^)					1.37 ± 0.89	
Lymphocyte (%)					25.19 ± 7.32	

According to the Montreal classification system. CRP, C-reactive protein.

### 2.2 DSS-induced colitis model in mice

NEAT1 knockout (NEAT1 KO) mice were produced at the Nanjing Institute of Biological Medicine in Jiangsu, China. The Institutional Animal Care and Use Committee of the Affiliated Hospital of Jining Medical University reviewed and approved all animal studies conducted. Subsequently, a colitis model induced by DSS was developed. In summary, WT C57BL/6 mice and NEAT1 KO mice (eight individuals in each group) were given 2.5% DSS (molecular weight, 36000–50000; MP Biomedicals, Solon, Ohio, USA) in their drinking water for a week, followed by two additional days of administration of sterile water. Daily observations were made to record the features of acute colitis, including diarrhea, the presence of bloody stools, changes in body weight, and survival rates. On the ninth day, all mice were euthanized, and colonic tissues were collected. A small segment of the colon (0.5 cm) was preserved in 4% paraformaldehyde for subsequent hematoxylin and eosin staining as well as immunofluorescence analysis. Another segment of the colon (0.5–1.0 cm) was utilized for RNA and protein extraction. Additionally, peripheral blood cells were harvested from the mice after lysis of red blood cells for flow cytometric evaluation.

### 2.3 Sequencing of gut microbiota

The gut microbiota sequencing process was conducted by Shanghai Ouyi Biotechnology Co., LTD. (Shanghai, China). Bacterial DNA was extracted from the feces of mice using the MagPure Soil DNA LQ Kit provided by Magan. The PCR amplification focused on the V3-V4 region of the bacterial 16S rRNA gene, employing the forward primer (5’-TACGGRAGGCAGCAG-3’) and the reverse primer (5’-AGGGTATCTAATCCT-3’). Following this, the PCR product was subjected to a second PCR round after being purified with AMPure XP beads. The purified product from the second round was quantified using Qubit, and its concentration was subsequently adjusted for sequencing purposes. Sequencing was carried out on the Illumina NovaSeq 6000 platform, generating 250 bp paired-end reads. Taxonomic annotation was performed using the SILVA v138 database. α-diversity and β-diversity analyses were conducted using QIIME2, and differential taxa were identified by LEfSe (LDA score >2.0, p<0.05).

### 2.4 Isolation and incubation of neutrophils

For the isolation of neutrophils from mouse bone marrow, bone marrow of both WT and NEAT1 KO mice was first harvested from the femurs and tibias of mice, after which red blood cells were lysed using red blood cell lysis buffer. Neutrophils were then separated utilizing the EasySep™ Mouse Neutrophil Enrichment Kit (STEMCELL, Canada). The isolated neutrophils were then resuspended in 1640 medium, enriched with 0.5% fetal bovine serum (FBS), and maintained at 37 °C in a 5% CO_2_ atmosphere. Purity of isolated neutrophils was confirmed by flow cytometry, resulting, with >85% Ly6G^+^CD11b^+^ cells.

### 2.5 Single-cell RNA-sequencing analysis

Colonic biopsies from individuals with UC and HCs were collected through colonoscopy for single-cell RNA sequencing (ScRNA-seq) analysis conducted by OE Biotech Co., Ltd in Shanghai, China. The generation of libraries for ScRNA-seq was accomplished utilizing the BD Rhapsody platform, followed by sequencing on the Illumina NovaSeq 6000 system. UMI tools facilitated the analysis of the single-cell transcriptome, which included identifying the whitelist of cell barcodes, extracting UMIs from those barcodes, and calculating expression counts per cell using the filtered clean fastq files. The Seurat54 software (version 4.0.0) was employed for normalizing and filtering cells, taking into account the percentage of mitochondrial (MT) genes and the minimum and maximum numbers of detected genes. For describing relationships among single cells, Uniform Manifold Approximation and Projection analysis was applied. To pinpoint marker genes within each cluster, the FindAllMarkers function in Seurat was utilized. Captured cells per sample ranged from 4, 500–7, 200, sequencing depth was approximately 50, 000 reads per cell, and low-quality cells were excluded if they had <200 genes detected or >15% mitochondrial gene expression. Additionally, differentially expressed genes were determined through the FindMarkers function in Seurat, using a significance threshold of p < 0.05 and |log2foldchange| > 0.58.

### 2.6 Analysis of NETs formation in neutrophils

Neutrophils were obtained from the bone marrow of both WT and NEAT1 KO mice, then exposed to LPS (300 ng/mL) for a duration of 3 hours. A total of 2 × 10^5 neutrophils were placed onto glass slides, subsequently fixed using 4% paraformaldehyde, and stained with Hoechst 33342 at a dilution of 1:1000 (Invitrogen, San Diego, CA, USA). The visualization of NETs was conducted using a confocal microscope (LSM 710; ZEISS, Oberkochen, Germany).

### 2.7 Determination of ROS levels in neutrophils

To determine the ROS levels produced by neutrophils, 2 × 10^6^ neutrophils were stimulated with LPS (300 ng/mL) for 3 h. Cells (1 × 10^4^) were collected and analyzed using an ROS assay kit (YEASEN, Shanghai, China). ROS probe (2’, 7’-dichlorodi-hydrofluorescein diacetate [DCFH-DA]; 10 μM) was added into the medium and incubated with the neutrophils for 30 min. Then, cells were washed thrice with and resuspended in cold phosphate-buffered saline (PBS) for flow cytometric analysis (DxFLEX; Beckman Coulter, Inc., USA). Emission wavelength of DCFH-DA was set at 530 nm.

### 2.8 Quantitative reverse transcription-polymerase chain reaction

Total RNA was extracted from the biopsies and cell samples using the TRIzol reagent (Invitrogen) and reverse-transcribed into cDNA using the 5 X All-In-One RT Master Mix (abm, Richmond, BC, Canada), according to the manufacturers’ instructions. RT-PCR was performed using the following conditions: 25 °C for 10 min and 42 °C for 15 min, followed by 85 °C for 5 min. The synthesized cDNA was stored at −20 °C. qRT-PCR was performed using SYBR green. All qRT-PCR analyses were performed in triplicates. All primers were synthesized by ShengGong BioTek (Shanghai, China), and glyceraldehyde 3-phosphate dehydrogenase (GAPDH) was used as an endogenous reference gene (**Table 2**).

**Table 2:** Human and mouse primers used to amplify the coding and adjacent non-coding sequences of genes.

Gene name	Forward primer	Reverse primer
NEAT1 (H)	GGC AGG TCT AGT TTG GGC AT	CCT CAT CCC TCC CAG TAC CA
GAPDH (H)	GGAGCGAGATCCCTCCAAAAT	GGCTGTTGTCATACTTCTCATGG
GAPDH (M)	AGGTCGGTGTGAACGGATTTG	GAGGAGCCATCCACAGTCTTCT
IL-1β (M)	GCAACTGTTCCTGAACTCAACT	ATCTTTTGGGGTCCGTCAACT
IL-6 (M)	TTGGTCCTTAGCCACTCCTTC	TAGTCCTTCCTACCCCAATTTCC
TNF-α (M)	CCCTCACACTCAGATCATCTTCT	GCTACGACGTGGGCTACAG
IFN-γ (M)	ATGAACGCTACACACTGCATC	CCATCCTTTTGCCAGTTCCTC
S100A8 (M)	AAATCACCATGCCCTCTACAAG	CCCACTTTTATCACCATCGCAA
S100A9 (M)	TCCATGATGTCATTTATGAGGGC	ATACTCTAGGAAGGAAGGACACC
CXCL10 (M)	CCAAGTGCTGCCGTCATTTTC	GGCTCGCAGGGATGATTTCAA
CCL2 (M)	TTCTCTGTACCATGACACTCTGC	CGTGGAATCTTCCGGCTGTAG
PFKM (M)	TGTGGTCCGAGTTGGTATCTT	GCACTTCCAATCACTGTGCC
Minpp1(M)	TCCCTGGTGTGATGTGTTTGA	ACCGGCTGTTAATGGTGTAGC
PKD1(M)	CTAGACCTGTCCCACAACCTA	GCAAACACGCCTTCTTCTAATGT
PKLR (M)	TCAAGGCAGGGATGAACATTG	CACGGGTCTGTAGCTGAGTG
SLC2A1 (M)	CAGTTCGGCTATAACACTGGTG	GCCCCCGACAGAGAAGATG
LDHC (M)	TTCACGGTGTAAGATTACTGTGG	TCGTATCAGCGTCAACAAGGG

### 2.9 Western blotting

Tissue and cell samples were lysed using the radioimmunoprecipitation assay lysis buffer supplemented with 10% protease and phosphatase inhibitors (Beyotime). Equal amounts of proteins were loaded, separated on a 10% sodium dodecyl sulfate-polyacrylamide gel, transferred to polyvinylidene fluoride membranes, blocked with 5% non‐fat milk for 2 h at room temperature, and incubated with primary antibodies overnight at 4°C. Antibody against citrullinated H3 (CitH3; 1:1000; ab5103; Abcam), p65(1:1000; 8242T; Cell Signaling Technology), or phosphorylated p65(p-p65, 1:1000; 3033S; Cell Signaling Technology) was used as primary antibody. Then, cells were incubated with HRP-conjugated secondary antibodies (1:3000; Beyotime Biotechnology) for 2 h at room temperature. The membranes were visualized in a dark room using an ECL reagent (Beyotime). Protein bands were quantified using the ImageJ software. Results were normalized to those of the GAPDH internal control.

### 2.10 Immunofluorescence

Neutrophils isolated from mouse bone marrow were centrifuged and resuspended in PBS, and 10 μL of cell suspension was applied to glass slides. Colon frozen sections and neutrophil slides were fixed with acetone for 20 min and blocked with 10% goat serum for 1.5 h. Tissues or cells were incubated overnight at 4 °C with the following primary antibodies against ZO-1 (1:100; 21773-1-AP; Proteintech), Occludin (1:100; ab216327; Abcam), E-cadherin (1:100; 20874-1AP; Proteintech), CD4 (1:200; ab133616; Abcam), F4/80 (1:200; ab300421; Abcam), MPO (1:100; AF3667; Biotechne), CitH3 (1: 200; ab5103; Abcam), or Ly6G (1: 200; RB6-8C5; Invitrogen). Samples were then incubated with Alexa Fluor^®^ 488- or 594-conjugated secondary antibodies in the dark for 60 min at room temperature, followed by counterstaining with DAPI. Images were acquired using a confocal laser scanning microscope.

### 2.11 RNA-seq analysis

Neutrophils were isolated from the bone marrow of mice exhibiting DSS-induced colitis, followed by the extraction of total RNA for RNA sequencing analysis (OE Biotech Co., Ltd). The total RNA preparations’ purity and concentration were assessed using a NanoDrop ND-2000 spectrophotometer. Transcriptome libraries were created with the VAHTS Universal V6 RNA-seq Library Prep kit and sequenced on a Lumina NovaSeq 6000 platform. The HISAT2 software was employed for comparison against the reference genome and for calculating gene expression, while HTSeq-count was utilized to determine the read counts for each gene. Principal component analysis and gene count visualization were conducted with R (version 3.2.0) in order to evaluate biological duplications in the samples. Differentially expressed genes were identified through DESeq2, applying a Q value threshold of <0.05 and fold changes > 2 or < 0.5. The enrichment analyses for Kyoto Encyclopedia of Genes and Genomes (KEGG) pathways and Gene Ontology (GO) were carried out using hypergeometric distribution within R (version 3.2.0).

### 2.12 Glycolytic rate assay

Neutrophils were seperated and purified from the bone marrow of WT and NEAT1 KO mice, and stimulated in presence or absence of LPS for 3h *in vitro*. Then, cells were harvested and seeded into a 96-well Seahorse plate at a density of 7 × 10^5/well in Seahorse XF assay medium. Glycolytic rate assay kit (Agilent, Santa Clara, CA) was used to determine extracellular acidification rate (ECAR) and glycolytic proton efflux rate (glycoPER). glycoPER represents glycolytic proton efflux rate, which directly reflects real-time glycolytic flux. Sequential injections of the mixture of rotenone and antimycin A (Rot/AA), and 2-Deoxy-Dglucose (2-DG) were performed to measure basal glycolysis, compensatory glycolysis, and post-2-DG acidification in neutrophils, following the manufacturer’s protocol.

### 2.13 Statistical analysis

Data are expressed as the mean ± standard error of the mean. Data were analyzed using GraphPad Prism. All experiments were conducted at least in triplicates. Comparisons between two groups were performed with an unpaired Student’s t-test. One-way ANOVA followed by Tukey’s multiple comparisons test or two-way ANOVA followed by Sidak’s multiple comparisons test was used for comparisons among multiple groups. P values were adjusted for multiple comparisons using the Benjamini-Hochberg false discovery rate (FDR) method where applicable. Spearman correlation analyses were used to explore associations between NEAT1 expression and UC-related clinical indicators, and data analyses were all done in R version 4.3.1. Statistical significance was set at p < 0.05.

## 3 Results

### 3.1 NEAT1 is upregulated in the inflamed colonic mucosa of patients with active UC

**Figure 1:**
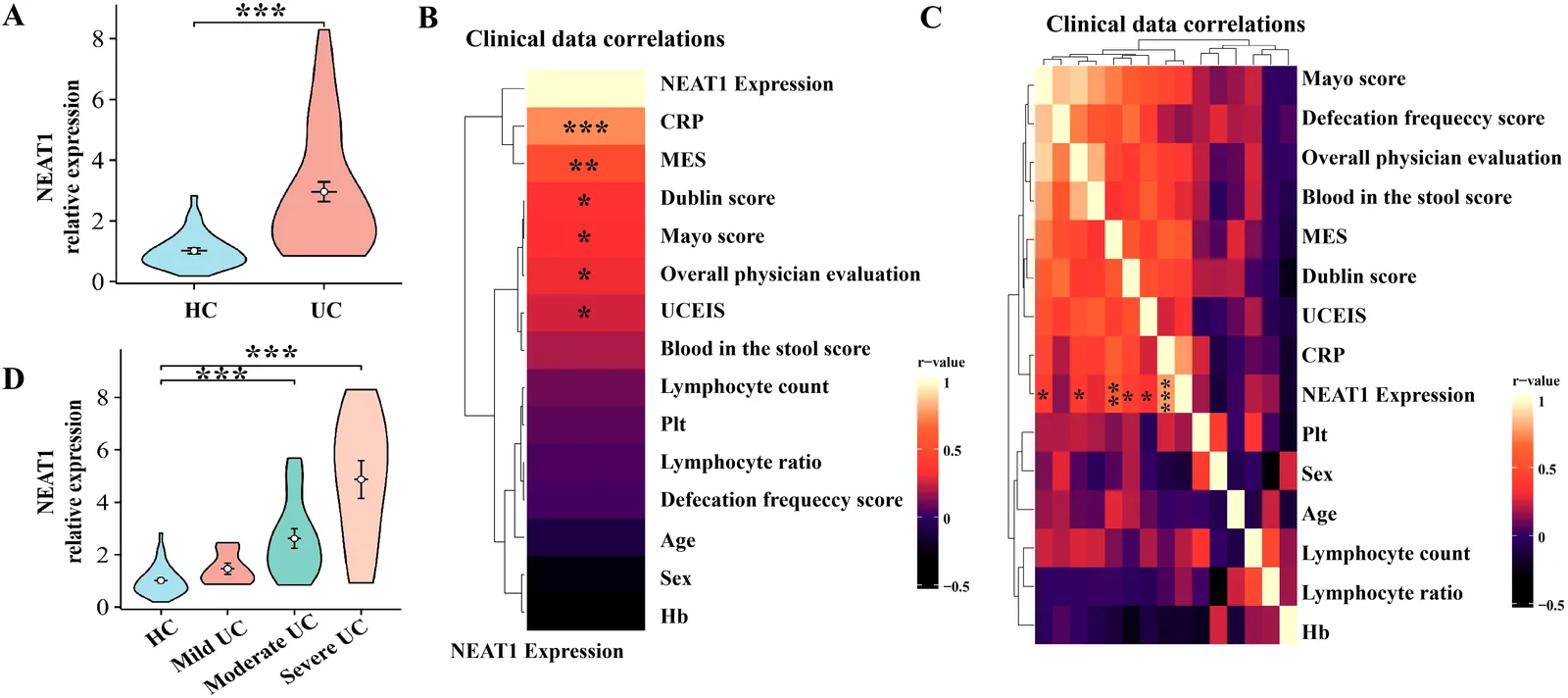
Expression of NEAT1 is elevated in inflamed colon mucosal biopsies of UC patients. **(A)** The levels of NEAT1 in the inflamed mucosa of patients with active UC and HCs were assessed using qRT-PCR. RNA was extracted from colon biopsy samples collected from HC individuals (n = 40), and those with active UC (n = 39). Gene expression was normalized to GAPDH in each group. ***p<0.001. **(B)** Spearman's correlation coefficient values were computed to analyze the relationship between NEAT1 expression in the intestinal mucosal tissues of UC patients and various clinical indicators. *p< 0.05, **p< 0.01, ***p < 0.001. **(C)** A heatmap illustrating the correlations between NEAT1 expression in intestinal mucosal tissue and clinical data for UC patients was generated. The Spearman correlation values pertaining to NEAT1 expression and clinical indicators were hierarchically clustered to reveal groups of closely related metrics. The axes in the analysis displayed each metric in a consistent order, with only the labels for the y-axis presented. *p< 0.05, **p< 0.01, ***p < 0.001. **(D)** NEAT1 expression in the colonic mucosa of patients categorized as having mild (n = 10), moderate (n = 16), and severe UC (n = 13), as well as HC (n = 40), was investigated using qRT-PCR. Gene expression was normalized to GAPDH in each group.**p< 0.01, ***p < 0.001.

To investigate the involvement of NEAT1 in the development of UC, we assessed the expression of NEAT1 in colonic biopsy samples from patients experiencing active UC. qRT-PCR results revealed that NEAT1 was considerably elevated in the intestinal mucosa of active UC patients when compared to HCs (**Figure 1A**). Furthermore, we examined the relationship between NEAT1 expression in the intestinal mucosa and various clinical parameters related to UC (including CRP, Hb, Plt, MES, Mayo score, UCEIS, Dublin score, among others). As shown in **Figures 1B, C**, a strong positive correlation was observed between NEAT1 expression and core clinical indicators such as serum CRP, MES, Dublin score, total Mayo score, overall physician assessment, and UCEIS in patients with UC. These clinical and endoscopic indices all reflect the severity of intestinal mucosal injury and systemic inflammatory burden, further supporting that elevated NEAT1 level is closely linked to aggravated disease activity in UC patients. To further investigate our hypothesis regarding the link between NEAT1 expression and disease activity in UC patients, we evaluated disease severity based on the Mayo score. Mild UC was classified as a Mayo score between 3 and 5, moderate UC as a score from 6 to 10, and severe UC as a score of 11 to 12. Notably, regarding the Mayo score, NEAT1 expression was most pronounced in the severe cohort, followed by those with moderate and mild forms of the disease (**Figure 1D**), which suggested a positive association between NEAT1 expression and UC disease activity. Collectively, these findings imply that NEAT1 is involved in the mechanisms of UC pathogenesis and may represent a potential therapeutic target for treating this condition.

### 3.2 NEAT1 deficiency alleviates DSS-induced colitis in mice

**Figure 2:**
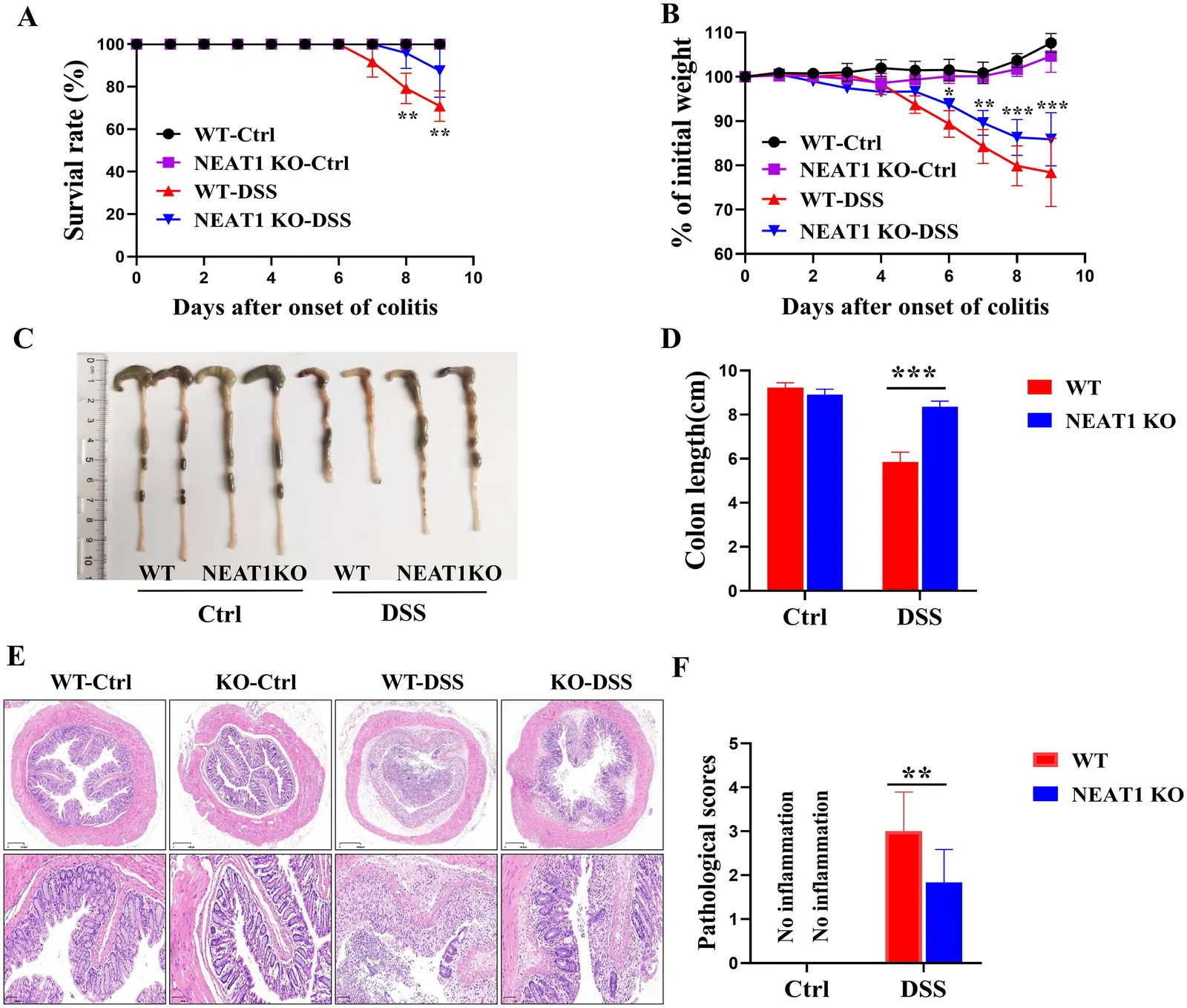
NEAT1 deficiency alleviates dextran sulfate sodium (DSS)-induced colitis in mice. WT and NEAT1 KO mice (n = 8 in each group) were given 2.5% DSS in drinking water for continuous 7 days and then followed with regular water for another 2 days. On Day 9, all mice were sacrificed. **(A)** The survival rate in both groups after DSS insult.**p < 0.01. **(B)** The changes in body weight were expressed as a percentage of the original weight at the start of the experiment during a period of 9-day observation. *p < 0.05, **p < 0.01, and ***p < 0.001. **(C)** Gross morphology of the colons when mice were sacrificed. **(D)** The statistical length of colons in different groups. ***p <0.001. **(E)** Representative H&E staining images of distal colonic sections (original magnification × 200). Scale bars=100 µm. **(F)** The changes of pathological scores from colonic sections were calculated as indicated. **p<0.01.

To investigate the function of NEAT1 in colonic inflammation, we created a mouse model of DSS-induced colitis involving both WT and NEAT1 KO mice. Observations depicted in **Figures 2A, B** illustrated that the survival rate for NEAT1 KO mice was considerably higher than that of WT counterparts during a 9-day monitoring period, with notably lower body weight loss. On the 9th day, all subjects were sacrificed, revealing that the colon length in NEAT1 KO mice was significantly greater than in WT mice (**Figures 2C, D**). Histological analyses further indicated that NEAT1 KO mice exhibited reduced pathological scores compared to WT mice following DSS treatment (**Figures 2E, F**).

**Figure 3:**
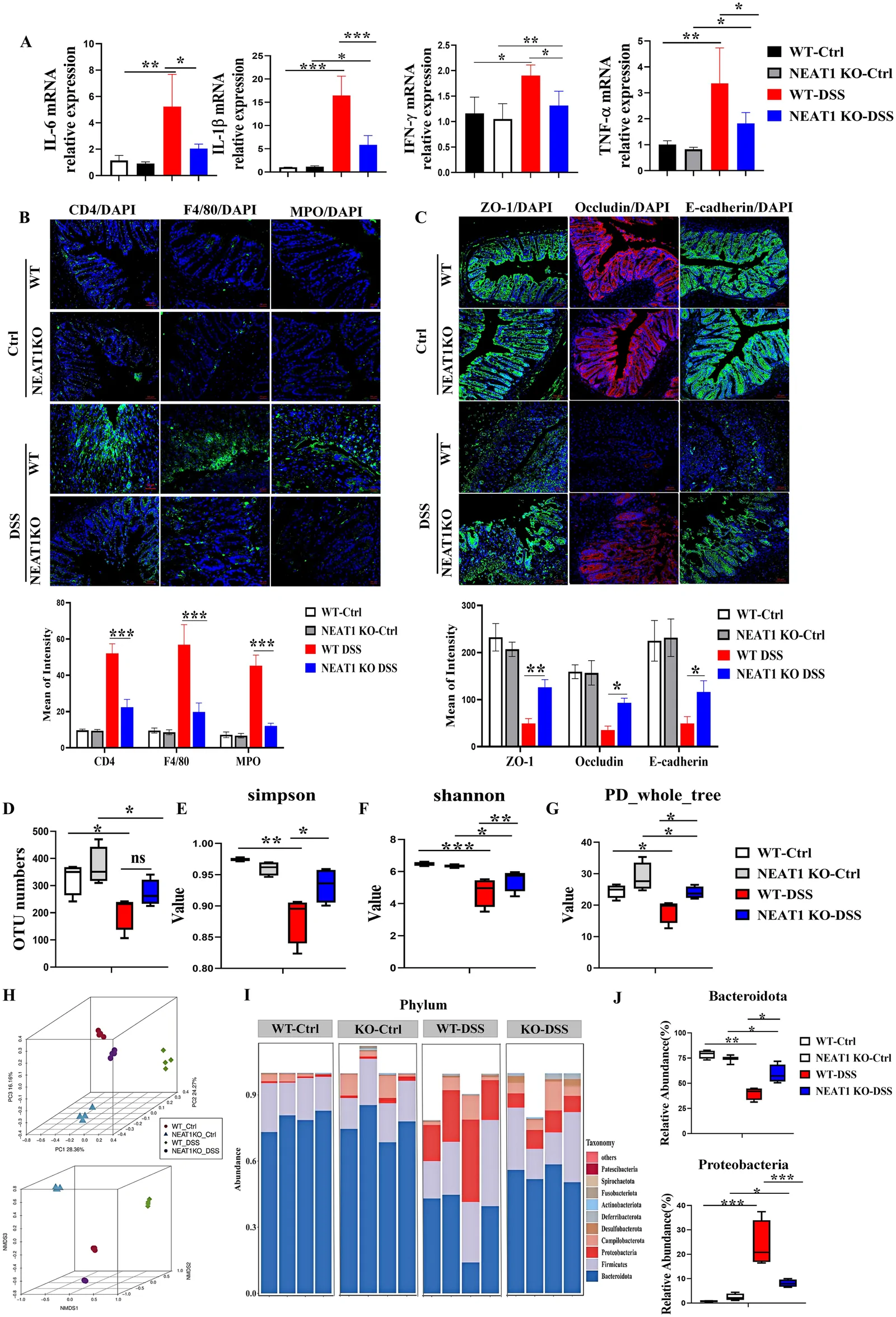
NEAT1 deficiency alleviates intestinal inflammation during colitis. WT and NEAT1 KO mice (n = 8 in each group) were given 2.5% DSS in drinking water for continuous 7 days and then followed with regular water for another 2 days. On Day 9, all mice were sacrificed, and the colonic tissues were collected. **(A)** The relative mRNA expressions of various pro-inflammatory genes (e.g., IL-6, IL-1β, IFN-γ, and TNF-α ) in colonic tissues from control and DSS-induced colitis mice were quantified by qRT-PCR. *p < 0.05, **p < 0.01, and ***p < 0.001. **(B)** Cellular fractions of CD4^+^ T cells, F4/80^+^ macrophages, and MPO^+^ neutrophils in colonic mucosa were determined by immunofluorescence. Scale bars = 20 μm. ***p < 0.001. **(C)** The expressions of tight junction proteins ZO-1, Occludin and E-cadherin were determined by immunofluorescence. Scale bars = 20 μm. *p < 0.05, and **p < 0.01. **(D-J)** The faeces were collected for 16S rRNA sequencing. **(D)** Operational taxonomic unit (OTU) numbers of intestinal flora in each group. The α-diversity of the microbiome among all groups depicted according to Simpson index **(E)**, Shannon index **(F)** and PD-whole-tree index**(G)**. *p < 0.05, **p < 0.01, and ***p < 0.001. **(H)** β-diversity analyses of the gut microbial were measured by principal coordinate analysis (PCoA, ray-Curtis distance) and non-metric multidimensional scaling (NMDS, Bray-Curtis distance) of mouse gut microbiota. **(I)** Phylum-level gut microbiota profile. **(J)** The relative abundance of *Bacteroidota* and *Proteobacteria*. *p < 0.05, **p < 0.01, and ***p < 0.001.

We also assessed the mRNA expression of pro-inflammatory mediators within the colon tissues across these groups. As depicted in **Figure 3A**, levels of IL-6, IL-1β, IFN-γ, and TNF-α were significantly lower in the colon tissues of the NEAT1 KO-DSS group. To further evaluate the extent of intestinal inflammation, we examined immune cell infiltration in the colonic lamina propria and assessed intestinal barrier integrity through immunofluorescent staining. We observed that the proportions of CD4^+^ T cells, F4/80^+^ macrophages, and MPO^+^ neutrophils were elevated in the DSS groups relative to the control groups. Furthermore, in contrast to the WT DSS group, NEAT1 KO DSS mice displayed reduced proportions of CD4^+^ T cells, F4/80^+^ macrophages, and MPO^+^ neutrophils (**Figure 3B**). Regarding intestinal barrier integrity, fluorescence staining for ZO-1, E-cadherin, and Occludin revealed that the absence of NEAT1 facilitated the restoration of tight junction proteins (**Figure 3C**).

Dysbiosis of intestinal microbiota significantly contributes to the pathogenesis of UC (30). We also conducted 16S rRNA sequencing to determine if the absence of NEAT1 could ameliorate intestinal microbiota dysbiosis. While there were no significant discrepancies in OTU counts between the WT DSS and NEAT1 KO DSS groups, the indices of α-diversity (e.g., Simpson, Shannon, and PD-whole tree) and β-diversity (PCoA and NMDS) all exhibited enhancements in the NEAT1 KO DSS group relative to the WT DSS group, highlighting the crucial influence of NEAT1 on the richness of the microbiome (**Figures 3D–H**). As for the bacterial composition and abundance, a significant decrease in *Bacteroidota* was observed in the DSS mice as compared to the control group, whereas an increase in *Proteobacteria* was noted, at the phylum level. Interestingly, in the NEAT1 KO-DSS group, the levels of *Bacteroidota* rose, and those of *Proteobacteria* fell compared to the WT-DSS group (**Figures 3I, J**). These findings strongly indicate that the absence of NEAT1 reduces inflammatory cells infiltration, enhances recovery of tight junction proteins, and alleviates intestinal microbiota dysbiosis in DSS-induced colitis mice.

### 3.3 NEAT1 is predominantly expressed in neutrophils and elevated in neutrophils from UC patients

**Figure 4:**
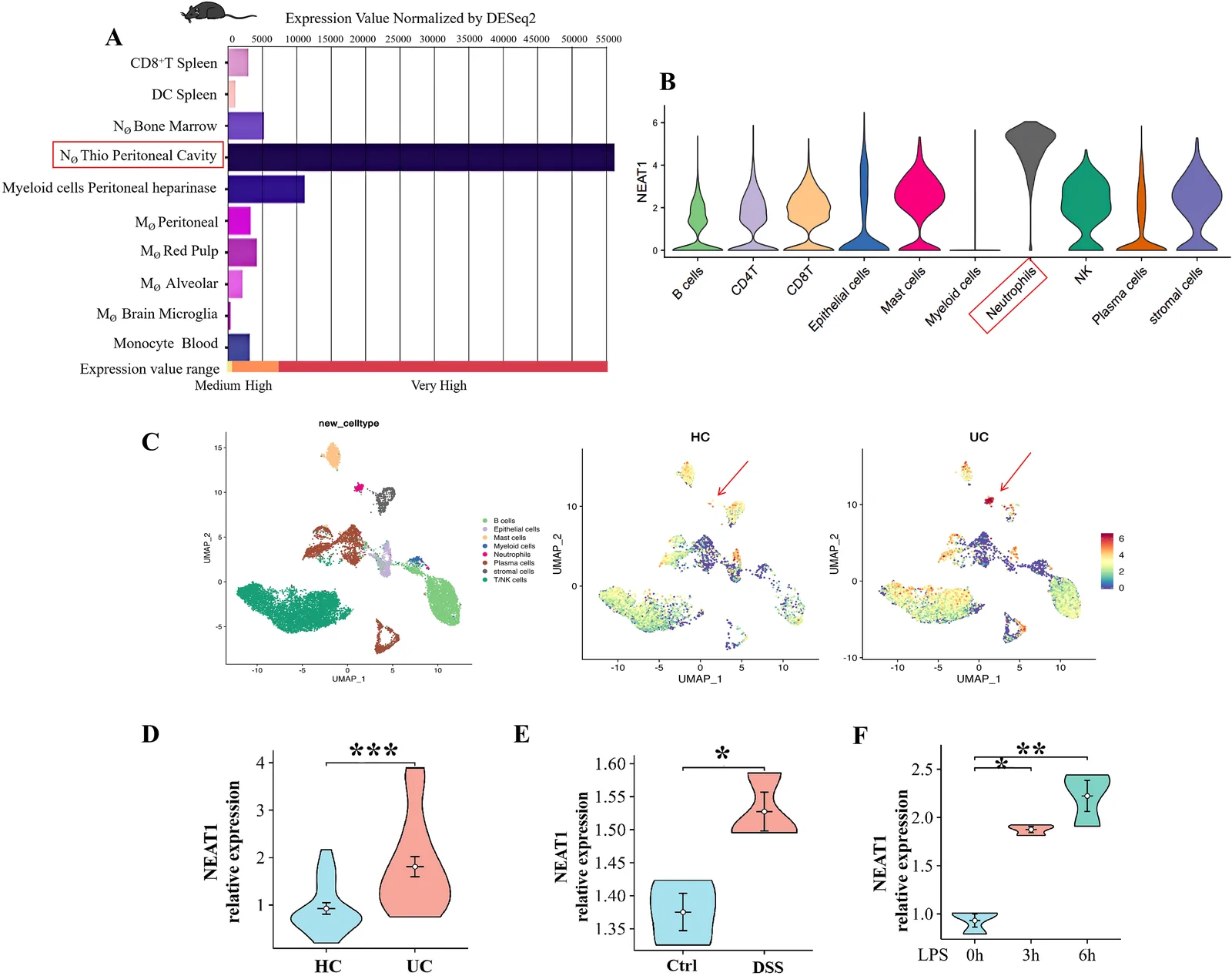
NEAT1 expression is highly increased on neutrophils from patients with UC and colitis mice. **(A)** NEAT1 expression analysis in immune cells from the Immunological Genome Project (ImmGen) bulkpopulation RNA-seq database. **(B)** Intestinal mucosal from UC patients and HC were analyzed by Single cell RNA Sequencing (ScRNA-Seq). NEAT1 expression in different cell subsets was visualized by Violin plot. **(C)** NEAT1 expression was increased on neutrophils from intestinal mucosa lamina propria of UC patients compared with that from HC. **(D)** Peripheral blood neutrophils were collected from UC patients (n = 21) and HC (n = 19), and expression of NEAT1 was detected by qRT-PCR. ***p < 0.001. **(E)** Bone marrow neutrophils were collected from WT and DSS-colitis mice (n=3 in each group), and NEAT1 expression was analyzed by RNA-Seq. *p < 0.05. **(F)** Bone marrow neutrophils were collected from WT and NEAT1 KO mice, stimulated with LPS, and NEAT1 expression of NEAT1 was detected by qRT-PCR. *p < 0.05, and **p < 0.01.

Previous research has shown that NEAT1 is broadly expressed across various immune cells, and mainly contributes to the pathogenesis of inflammatory immune diseases by modulating immune cell functions. We began our analysis by investigating NEAT1 levels in distinct types of immune cells, utilizing data from the Immunological Genome Project (31). Our analysis revealed that NEAT1 expression is more pronounced in neutrophils (NØ) in comparison to other immune cell types, including macrophages (MØ), dendritic cells (DC), CD8^+^T cells, and monocytes (**Figure 4A**). Additionally, we observed that peritoneal neutrophils induced by thioglycolate exhibited greater NEAT1 expression compared to neutrophils derived from bone marrow, suggesting an upregulation of NEAT1 during neutrophil activation. During the development and advancement of UC, immune cells located in the intestinal mucosal lamina propria play a critical role. Therefore, we conducted a further analysis of NEAT1 expression in immune cells from the lamina propria of UC patients and healthy controls using ScRNA-seq techniques, as outlined in our earlier study (32). As illustrated in **Figure 4B**, NEAT1 was present in multiple immune cell types, such as B cells, T cells, epithelial cells, neutrophils, NK cells, and mast cells, with particularly high levels observed in neutrophils. Furthermore, the expression of NEAT1 was significantly greater in lamina propria neutrophils from UC patients compared to those from HCs (**Figure 4C**).

Moreover, we isolated neutrophils from the peripheral blood of both UC patients and HCs for qRT-PCR analysis, which confirmed that NEAT1 expression in neutrophils from UC patients was markedly increased relative to that in HCs, aligning with the findings from the ScRNA-seq analysis (**Figure 4D**). We also explored NEAT1 expression in bone marrow neutrophils from WT mice and DSS-colitis mice by RNA sequencing, and the results showed that compared to bone marrow neutrophils from WT mice, NEAT1 expression was higher in bone marrow neutrophils from DSS-colitis mice (**Figure 4E**). In addition, under *in vitro* LPS condition, NEAT1 expression in bone marrow neutrophils from WT mice was increased remarkably (**Figure 4F**). These findings suggest that NEAT1 may participates the mucosal inflammation associated with UC via modulating neutrophils immune responses; however, the exact mechanisms involved warrant further research.

### 3.4 NEAT1 enhanced pro-inflammatory functions of neutrophils *in vitro*

**Figure 5:**
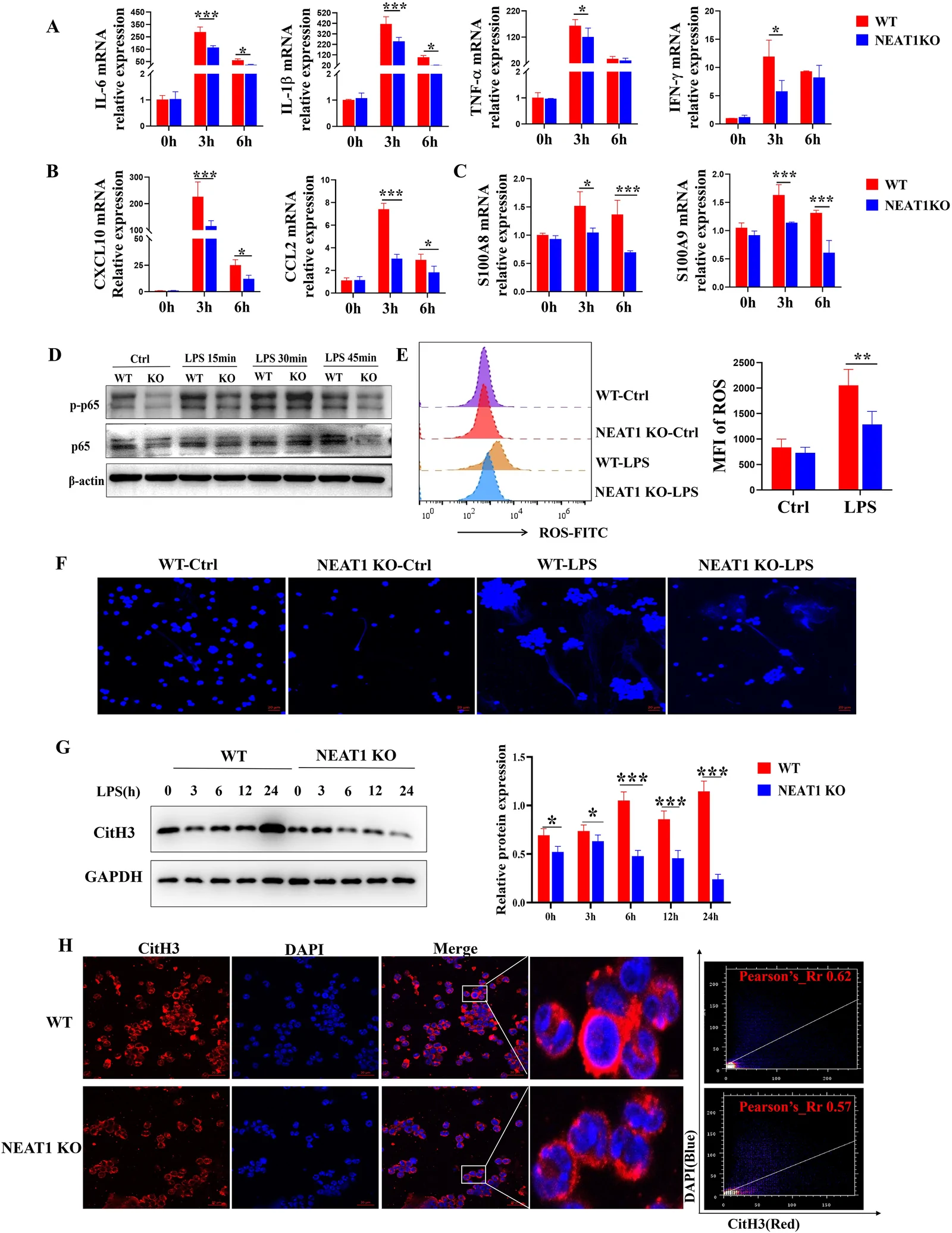
NEAT1-deficient neutrophils display impaired pro-inflammatory functions *in vitro*. Neutrophils were isolated from the bone marrow of NEAT1 KO mice and WT mice, and pre-treated *in vitro* with LPS for 3 h. Cells were then collected and relative mRNA expression of pro-inflammatory mediators were analyzed for IL-6, IL-1β, TNF-α, and IFN-γ **(A)**, CCL2 and CXCL10 **(B)**, S100A8 and S100A9 **(C)**, respectively, by qRT-PCR. **p < 0.01, ***p < 0.001. **(D)**Western blot analysis of phosphorylation P65 (p-P65) and total p65 levels in neutrophils from WT and NEAT1 KO mice. β-actin served as a loading control. **(E)** Measurement of ROS production using flow cytometric analysis, and mean fluorescence intensity of ROS were statistically analyzed. **p < 0.01. **(F)** The formation of NETs was stained with Hoechst 33342, and examined by confocal microscopy (× 63). Scale bars = 20 μm. **(G, H)** CitH3 protein expression was detected by Western blotting **(G)**, and immunofluorescence **(H)**. Scale bars = 20 μm. The numbers per condition for experiments are 3.

During colitis, an excessive accumulation of neutrophils in the inflamed intestine can lead to the production of various pro-inflammatory mediators, thus contributing to the persistence and intensification of intestinal inflammation. However, the influence of NEAT1 on the modulation of pro-inflammatory mediators by neutrophils in UC is not yet fully understood. In pursuit of this understanding, neutrophils were isolated from the bone marrow of both WT and NEAT1 KO mice, then stimulated with LPS (300 ng/mL) for 3 hours, followed by qRT-PCR analysis to assess levels of pro-inflammatory cytokines and chemokines. As depicted in **Figures 5A, B**, the mRNA expression of pro-inflammatory cytokines, including IL-1β, IL-6, TNF-α, and IFN-γ, alongside chemokines such as CCL2 and CXCL10, were significantly reduced in bone marrow neutrophils derived from NEAT1 KO mice. Additionally, the expression of calprotectins (S100A8 and S100A9), known markers of UC relapse, were also notably diminished in neutrophils lacking NEAT1 (**Figure 5C**). Previous studies have shown that the NEAT1-NF-κB axis is highly relevant in intestinal inflammation (28). We assessed NF-κB activation by measuring p-p65/p65, and the results showed that NF-κB signaling was suppressed after NEAT1 knockdown (**Figure 5D**).

It has been documented that abnormal and prolonged release of ROS and the formation of NETs can directly impact mucosal injury in UC (33). **Figure 5E** illustrate that ROS levels were also decreased in neutrophils from NEAT1 KO mice compared to those from WT mice following LPS stimulation. The generation of ROS is a critical step in NETs formation, which aligns with our findings of reduced NETs formation observed through Hoechst 33342 staining in neutrophils from NEAT1 KO mice compared to their WT counterparts post-LPS stimulation (**Figure 5F**). Furthermore, we assessed the protein expression of CitH3, a marker indicative of NETs formation, via western blotting and immunofluorescence. Similarly, the expression of CitH3 protein in neutrophils was found to be compromised after the knockout of NEAT1 (**Figures 5G, H**). Consequently, NEAT1 appears to play a significant role in enhancing the pro-inflammatory capacities of neutrophils.

### 3.5 NEAT1-deficient neutrophils show weakened pro-inflammatory functions in colitis mice

**Figure 6:**
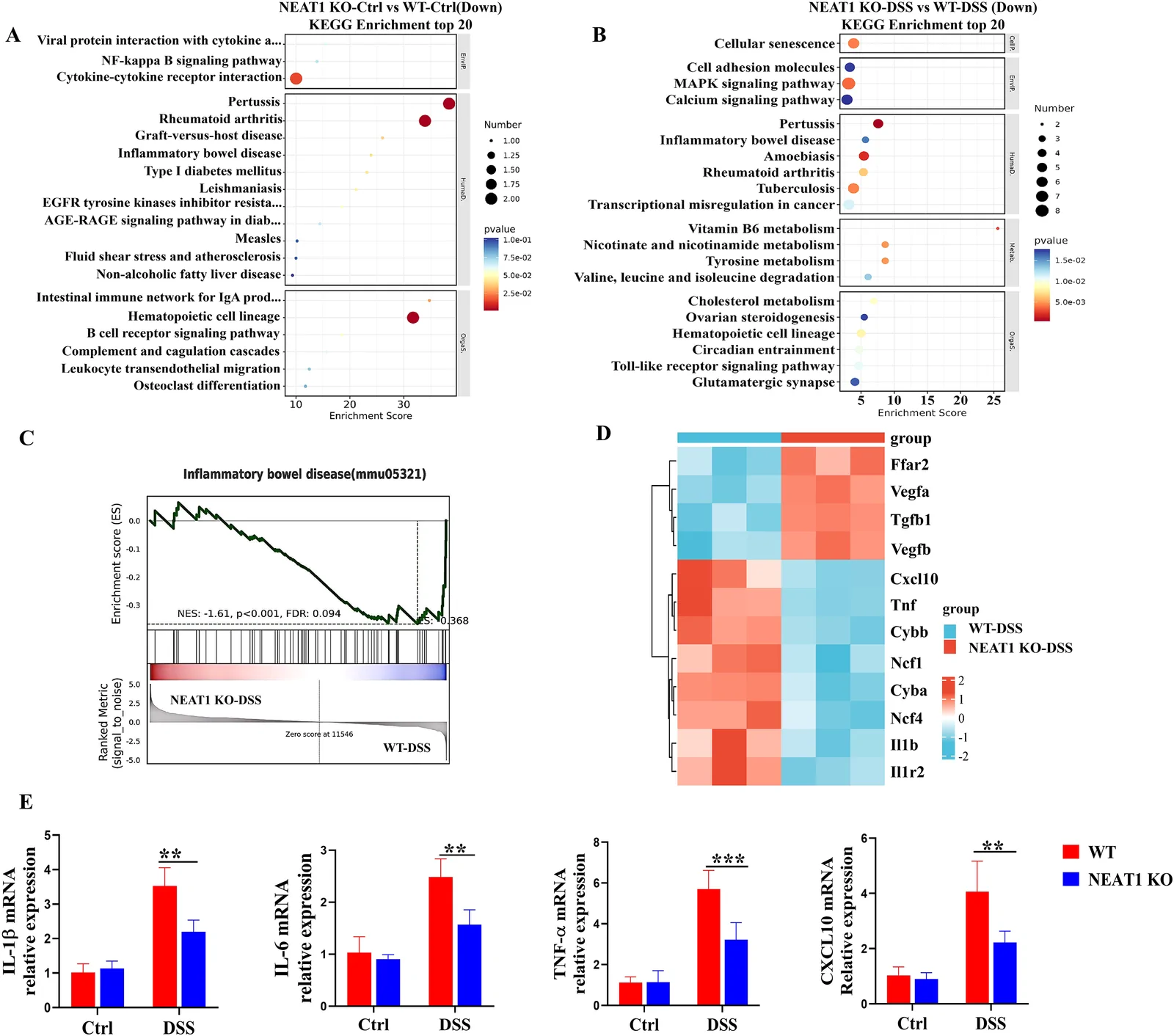
NEAT1-deficient neutrophils show weakened pro-inflammatory genes in colitis. Bone marrow neutrophils were isolated from the untreated and DSS groups of WT- and NEAT1 KO mice. **(A, B)** RNA-seq analysis was performed, and KEGG pathway analysis of the differentially down expressed genes. **(C)** Gene set enrichment analysis showing enrichment of genes involved in inflammatory bowel disease. **(D)** Heatmap of differentially expressed genes between control and NEAT1-deficient neutrophils related to inflammation. **(E)** qRT-PCR was used to quantify the mRNA expression of some differentially expressed genes. **p < 0.01, and ***p < 0.001.

Considering that NEAT1 is predominantly expressed in neutrophils and enhances their pro-inflammatory functions *in vitro*, we conducted further investigation into the impact of NEAT1 on neutrophil functionality *in vivo*. We isolated bone marrow neutrophils from both untreated and DSS mice, subsequently performing RNA-Seq analysis. KEGG analysis of downregulated differentially expressed genes in neutrophils of WT and NEAT1 KO mice in the control group and DSS group revealed that genes with decreased expression were associated with inflammatory bowel disease (**Figures 6A, B**). This association was further substantiated through gene-set enrichment analysis (**Figure 6C**). We then concentrated on inflammation- and IBD-related genes as indicated by our heatmap analysis and discovered that the absence of NEAT1 significantly reduced the expression levels of Il1b, Cxcl10, Tnf, Tgfb1, Cyba, Cybb, Ncf1, and Ncf4 in neutrophils (**Figure 6D**), some of which were validated through qRT-PCR (**Figure 6E**).

**Figure 7:**
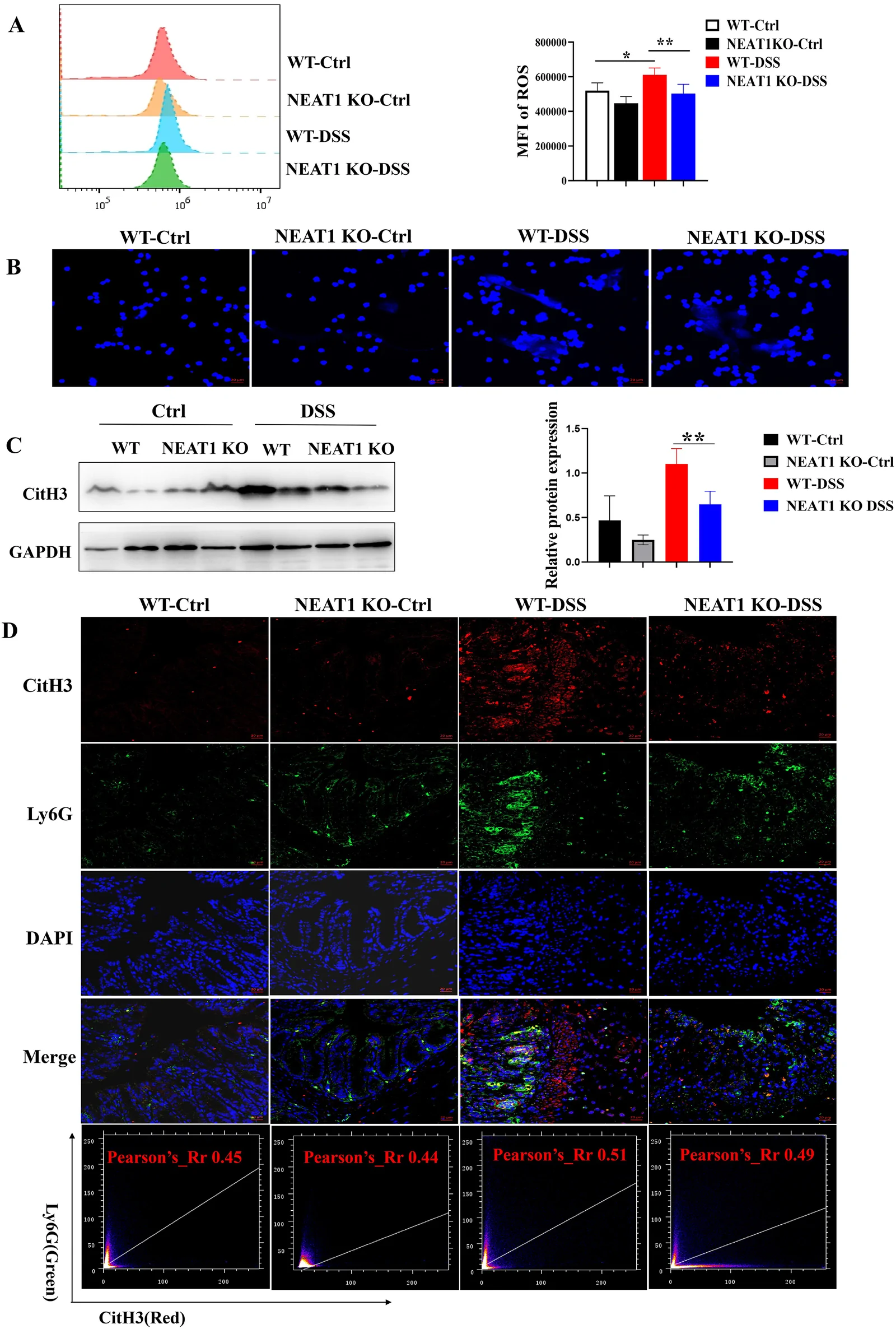
NEAT1-deficient neutrophils show reduced ROS and NETs production in colitis. Neutrophils were isolated from the bone marrow of WT- and NEAT1 KO-DSS mice. **(A)** Flow cytometric analysis was used to measure the ROS production, and mean fluorescence intensity of ROS were statistically analyzed. *p < 0.05, **p < 0.01. **(B)** The formation of NETs was stained with Hoechst 33342, and examined by confocal microscopy (× 63). Scale bars = 20 μm. **(C)** CitH3 protein expression was detected by Western blotting. **(D)** Colon tissues were collected from mice, and CitH3 expression was detected by immunofluorescent. Scale bars = 20 μm. The numbers per condition for experiments are 3.

Additionally, we observed that genes involved in the production of ROS in neutrophils, specifically Cyba, Cybb, Ncf1, and Ncf4, were all downregulated in the NEAT1 KO-DSS group. Thus, we further analyzed ROS production in bone marrow neutrophils obtained from DSS mice. Our findings indicated that neutrophils from the NEAT1 KO-DSS group had a lower ROS production level compared to those from the WT-DSS group (**Figure 7A**). Furthermore, we assessed NETs formation through Hoechst 33342 staining and the protein expression of CitH3 in neutrophils via western blotting, revealing that NETs formation was suppressed in neutrophils from NEAT1 KO-DSS group than that from the WT-DSS group, and CitH3 protein level was reduced in neutrophils from the NEAT1 KO-DSS group compared to the WT-DSS group (**Figures 7B, C**), which were consistent with our *in vitro* findings. We also observed a marked decrease in CitH3 expression in the colon tissue sections of NEAT1 KO-DSS mice compared to those of the WT-DSS mice via immunofluorescence staining (**Figure 7D**).

### 3.6 NEAT1-deficient neutrophils show a metabolic shift towards glycolysis in colitis mice

**Figure 8:**
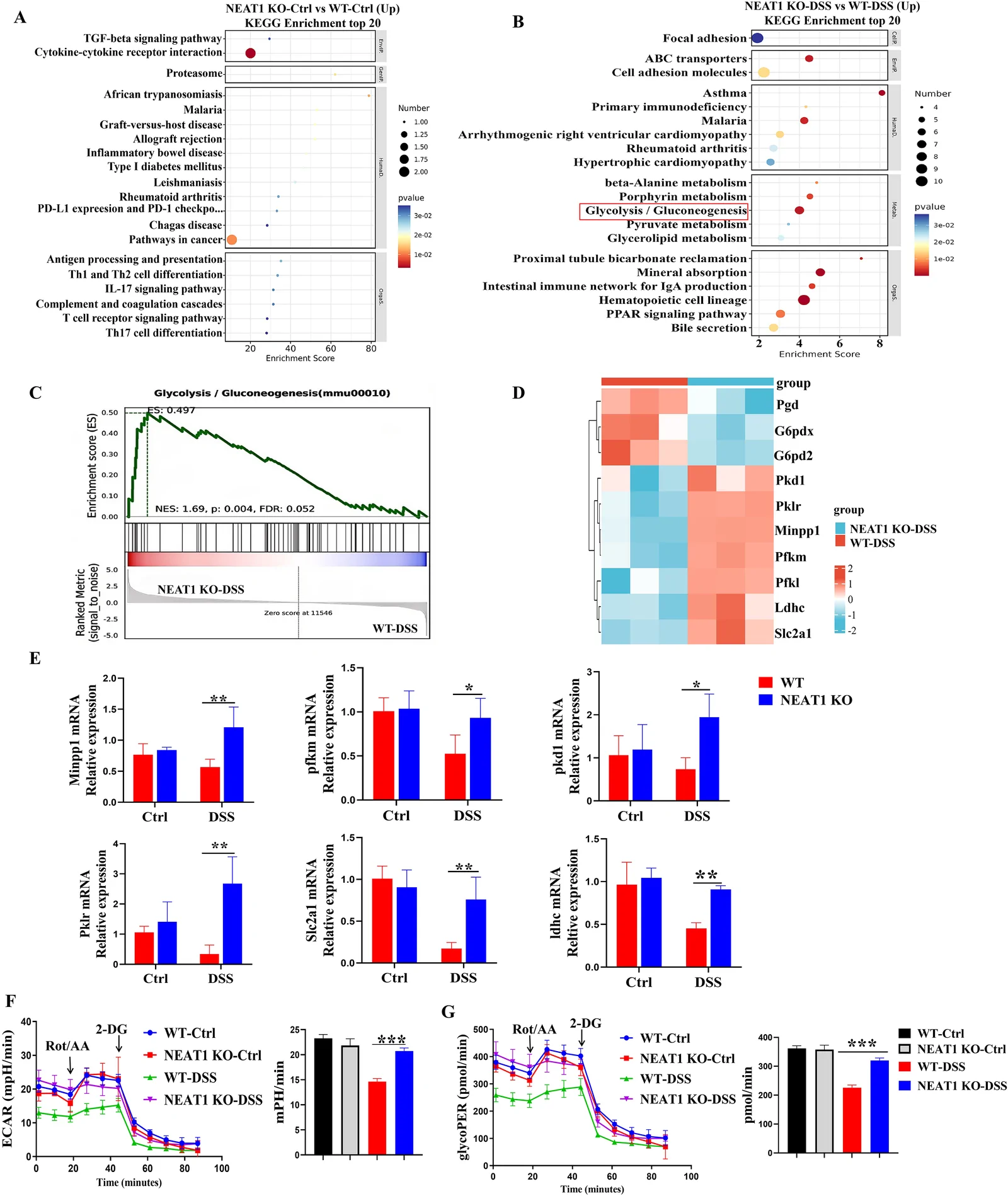
NEAT1-deficient neutrophils show a metabolic shift towards glycolysis in colitis. Bone marrow neutrophils were isolated from the untreated and DSS groups of WT- and NEAT1 KO mice. **(A, B)** RNA-seq analysis was performed, and KEGG pathway analysis of the differentially up expressed genes. **(C)** Gene set enrichment analysis showing enrichment of genes involved in glycolysis. **(D)** Heatmap of differentially expressed genes between control and NEAT1-deficient neutrophils related to glycolysis. **(E)** qRT-PCR was used to quantify the mRNA expression of some differentially expressed genes. *p < 0.05, and **p < 0.01. **(F, G)** glycolytic flux were measured using Seahorse XF assay, and ECAR **(F)** and glycoPER **(G)** were statistically analyzed. ***p < 0.001.

While the transcriptome data revealed broad suppression of pro-inflammatory transcriptional signatures in NEAT1-deficient neutrophils, pathway enrichment analysis further uncovered prominent metabolic reprogramming characterized by significantly upregulated glycolysis-related gene clusters (**Figures 8A, B**). This correlation was corroborated by gene-set enrichment analysis (**Figure 8C**). We concentrated on the glycolysis-related genes highlighted by the heatmap analysis, discovering that the absence of NEAT1 increased the expression of Minpp1, pfkl, pfkm, pkd1, pklr, Ldhc, and Slc2a1 (**Figure 8D**), of which some were validated through qRT-PCR (**Figure 8E**). Consequently, we further evaluated the dynamic alterations in glycolysis within the neutrophils sourced from the bone marrow of DSS-colitis mice, utilizing the Seahorse XF glycolytic rate assay. Remarkably, neutrophils from the NEAT1 KO-DSS group displayed elevated levels of ECAR and glycoPER in comparison to those from the WT-DSS group (**Figures 8F, G**), revealing an increased glycolysis that aligns with the RNA-seq outcomes. These results indicate a rise in glycolytic activity in NEAT1-deficient neutrophils derived from the colitis mouse model.

### 3.7 NEAT1 modulates ROS and NETs formation of neutrophils via glycolysis

**Figure 9:**
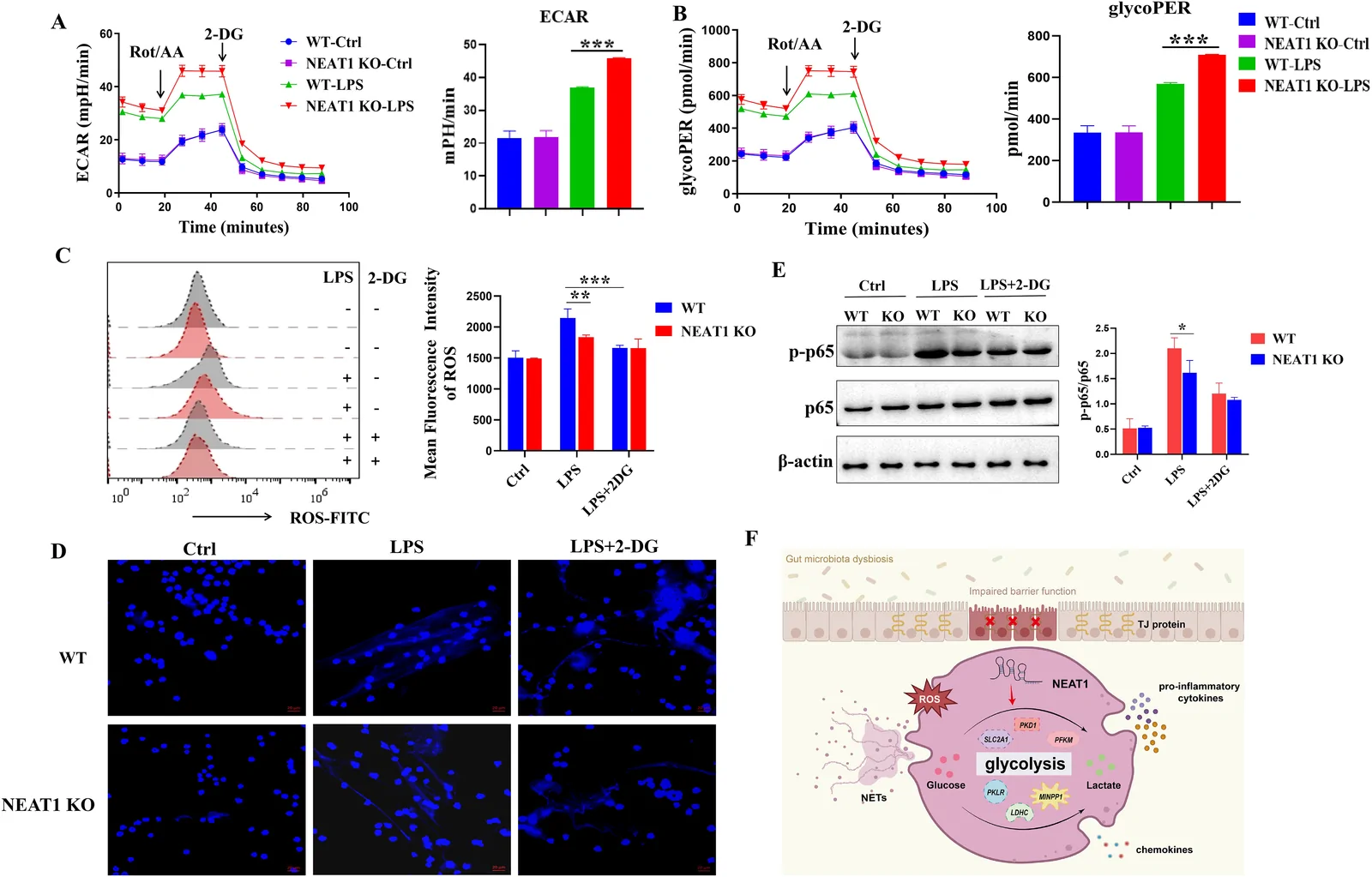
NEAT1 modulates ROS and NETs formation of neutrophils via glycolysis. **(A, B)** The bone marrow neutrophils from WT and NEAT1 KO mice were isolated, and stimulated with LPS for 3 h. Real-time changes in ECAR **(A)** and glycoPER **(B)** were detected using Seahorse XF assay. **(C–E)** NEAT1-deficient neutrophils were pretreated with 1 mM 2-DG for 30 min and then incubated with LPS for 3 h. ROS production was measured by flow cytometric, and mean fluorescence intensity of ROS were statistically analyzed **(C)**; NETs formation was stained with Hoechst 33342, and examined by confocal microscopy (× 63). Scale bars = 20 μm **(D)**; Western blot analysis of p-P65 and total p65 levels in neutrophils from WT and NEAT1 KO mice. β-actin served as a loading control **(E)**. **(F)** Model illustrating our findings. See the text for details.

To further explore the alterations in glycolysis associated with neutrophil activation, we stimulated bone marrow neutrophils harvested from WT and NEAT1 KO mice with LPS for 3 h, assessing real-time fluctuations in ECAR and glycoPER using a Seahorse system. As illustrated in **Figures 9A, B**, following 3 h of LPS treatment, neutrophils lacking NEAT1 demonstrated elevated levels of ECAR and glycoPER compared to control neutrophils after LPS treatment, which aligns with *in vivo* observations from neutrophils in DSS-colitis mice.

Glycolysis is recognized as the primary metabolic pathway in neutrophils, playing a vital role in the regulation of cellular functions (34). Subsequently, we investigated the involvement of glycolysis in the regulation of neutrophil immune functions by NEAT1. We exposed NEAT1-deficient bone marrow neutrophils to 2-Deoxy-Dglucose (2-DG), a glycolysis inhibitor, either in its presence or absence, and found that the application of 2-DG enhanced the production of ROS and the formation of NETs, which were diminished due to NEAT1 deficiency (**Figures 9C, D**). Interestingly enough, our study revealed that treatment with 2-DG suppresses p65 phosphorylation, and the difference between the WT group and the NEAT1 KO group is abolished, preliminarily demonstrating glycolysis functions upstream of NEAT1-mediated NF-κB activation in neutrophils (**Figure 9E**). These findings suggest that the lack of NEAT1 alters neutrophil functions, such as ROS production and NETs formation, at least partially by promoting glycolysis.

## 4 Discussion

UC, a specific type of IBD, is characterized by the development of neutrophilic cryptitis and the presence of crypt abscesses. Neutrophils play a crucial role in both the onset and progression of UC. When neutrophils malfunction, it can lead to abnormal immune responses in UC, suggesting that finding a balance between their protective and harmful functions could offer new therapeutic strategies for managing this condition (35). While previous studies have linked NEAT1 to the pathogenesis and progression of UC (23–28), its specific role in regulating neutrophil behavior in the context of UC remains poorly understood. In our research, we explored the significance and mechanisms by which NEAT1 influences neutrophil functions during UC. Our findings revealed that NEAT1 is predominantly expressed in neutrophils and that higher levels of NEAT1 in colonic mucosal tissues correlate positively with UC disease activity. NEAT1 downregulation leads to elevated neutrophil glycolysis, reduced ROS, NETs and pro-inflammatory factors, and ultimately relief of intestinal mucosal inflammation (**Figure 9F**).

NEAT1 is a long non-coding RNA (lncRNA) derived from chromatin, showing extensive expression in numerous immune cell types and playing a critical role in inflammatory reactions (36–38). In a murine model of experimental autoimmune uveitis, it has been demonstrated that NEAT1 facilitates antigen-specific Th17 cell responses by targeting NonO and miR-128-3p (39). Within macrophages, NEAT1 promotes the polarization of the M1 phenotype by influencing the miR-188-5p/BTK axis (20). Its expression is also positively associated with the proportions of Th1 and Th17 cells in patients with sepsis (40). In this research, we discovered a notable increase in NEAT1 expression within the intestinal mucosa of patients experiencing active UC, which showed a positive correlation with the severity of the disease. Furthermore, NEAT1 knockout mice exhibited reduced intestinal mucosal inflammation following DSS exposure, characterized by decreased infiltration of inflammatory cells, improved recovery of tight junction proteins, and a correction of intestinal microbiota imbalances. Additional investigations revealed that NEAT1 was primarily expressed in neutrophils from both the lamina propria and the peripheral blood of patients with UC.

Neutrophils represent the most prevalent type of white blood cell in humans and are recognized for their dual roles in promoting mucosal healing while also exerting strong pro-inflammatory responses during UC. The pro-inflammatory cytokines and chemokines produced by neutrophils can facilitate the onset and advancement of mucosal inflammation by drawing other immune cells to the site of inflammation in the mucosa (41). In our present research, we observed that the production of neutrophil-derived pro-inflammatory cytokines (such as IL-6, IL-1β, TNF-α, and IFN-γ) and chemokines (including CXCL10 and CCL2) was significantly reduced in neutrophils lacking NEAT1. Calprotectin, which is a heterodimer comprising S100A8 and S100A9, constitutes roughly 40% of the cytosolic proteins found in neutrophils and is known to play a role in the development of UC (42). Calprotectin concentrations are significantly increased in both stool and plasma of individuals with UC and show a positive correlation with the severity of the disease (43). Our findings indicated that neutrophils deficient in NEAT1 produced lower amounts of S100A8 and S100A9. It’s worth noting that we used LPS as *in vitro* stimulus of neutrophils. Although it has always been regarded as a canonical and reliable immune stimulus derived from the outer membrane of gram-negative bacteria to elicit innate immune responses (44), it does not fully recapitulate the pathophysiological complexity of UC. Future studies should evaluate NEAT1 under additional disease-relevant stimuli, such as TNF-α, chemokines, or ROS-inducing conditions, to better define its regulation in UC-associated inflammation.

Accumulating evidence indicates that IBD is tightly associated with ROS buildup within colonic inflammatory lesions (45–47). Mucosal ROS levels in intestinal biopsies from patients with IBD are markedly increased, with concentrations at inflamed lesions reaching 10-100-fold those observed in healthy mucosa (48). Excessive build-up of ROS mediate diverse pathogenic mechanisms in UC. ROS directly oxidizes tight junction proteins and epithelial lipids to break intestinal barrier integrity (49). ROS reshapes gut microenvironment by elevating luminal electron acceptors to favor the expansion of pathogenic Proteobacteria (50). Aberrant formation of NETs is crucial in maintaining mucosal inflammation in UC (51). NETs can disrupt the function of the epithelial barrier and increase the likelihood of thrombotic events in UC (32, 52). Notably, ROS synergizes with NETs to amplify mucosal inflammatory cascade and aggravate crypt damage (53). Our results demonstrate that NEAT1 loss restrains neutrophil ROS overproduction via boosting glycolysis, thereby blocking the above ROS-driven pathogenic loops.

Compelling evidence indicates a significant interaction between neutrophils and gut microbiota concerning intestinal inflammation. Prolonged inflammatory stimulation induces massive neutrophil infiltration and subsequent release of cytotoxic mediators, thereby remodeling the intestinal microenvironment. We found that genetic deletion of NEAT1 suppresses glycolytic reprogramming in neutrophils, which further blunts the release of ROS from mucosal infiltrating neutrophils. Intestinal ROS is a critical source of luminal nitrate (54), and leads to an increase in electron acceptors within the intestinal lumen, promoting the proliferation of pathogenic Proteobacteria (55, 56). Reduced ROS restrains the expansion of *Proteobacteria*, while creating a favorable niche for the colonization of anti-inflammatory commensal *Bacteroidota*, ultimately reversing colitis-associated gut dysbiosis observed in NEAT1 KO mice. Conversely, the intestinal microbiota can also influence neutrophil recruitment, activation, and functionality. A more recent study highlighted that *Bacteroides uniformis* could suppress neutrophil infiltration and diminish the formation of NETs, thereby mitigating pancreatic damage (57). Interestingly, our findings demonstrated that the lack of NEAT1 led to an increase in *Bacteroides* richness in the flora while also reducing NETs formation in neutrophils. Above all, our study suggest a potential immune-microbe regulatory network underlying NEAT1-mediated intestinal inflammation.

To carry out effector functions, neutrophils depend on straightforward metabolic routes. Although glycolysis is the predominant metabolic pathway in neutrophils, recent investigations have uncovered additional metabolic routes within these cells, including the pentose-phosphate pathway (PPP) and the citric acid cycle (34). The PPP plays a crucial role in generating NADPH (58), which is subsequently oxidized by NADPH oxidase to produce ROS and ultimately lead to the formation of NETs (59). A shift in the metabolic pathway from PPP to glycolysis could result in diminished ROS and NETs production in neutrophils. Prior research demonstrated that cyclosporine A enhances glycolysis while inhibiting ROS release in neutrophils from patients with acute severe UC (60). Selective activation of PFKL blocks the transition from glycolytic pathways to PPP in PMA-activated neutrophils, leading to reduced ROS production and NETs formation (61). Consistent with the findings from the aforementioned studies, we observed that NEAT1-deficient neutrophils exhibited a marked increase in glycolytic activity, alongside a decrease in ROS and NETs release. Additionally, we noted that the levels of G6pd2, G6pdx, and pgd, which are known to influence G6PD activity (62), were significantly downregulated in NEAT1-deficiency neutrophils. Given that G6PD is an essential enzyme in the PPP, we hypothesize that neutrophils lacking NEAT1 may experience reduced PPP efficacy. Furthermore, the use of 2-DG to inhibit glycolysis significantly enhanced the production of ROS and NETs, a process that was inhibited by NEAT1. This evidence strongly indicates that deleting NEAT1 plays a crucial role in mitigating excessive mucosal inflammation during intestinal colitis by supporting neutrophil glycolysis, which in turn limits the overactivation of neutrophils.

Notably, the expressions of inflammatory cytokines show inconsistent trends after the alteration of glycolysis (data not shown), indicating that the regulatory mechanism of NEAT1 on neutrophils may also involve other pathways besides glycolysis. In addition, direct measurements of PPP flux, or G6PD activity were not performed in the present study, which represents an important limitation. Future studies using targeted metabolomic analyses will be necessary to formally confirm the metabolic redistribution between glycolysis and PPP underlying NEAT1-mediated neutrophil functional regulation. Our current data merely support a functional association between NEAT1 deficiency, altered glycolytic phenotype, and neutrophil effector responses. The specific molecular mechanism by which NEAT1 regulates the glycolysis of neutrophils still requires further in-depth exploration.

In conclusion, our findings indicate that the knockout of NEAT1 is essential for regulating excessive pro-inflammatory responses, facilitating the restoration of the intestinal mucosal barrier, and enhancing the balance of intestinal microbiota during the progression of UC. Mechanistically, the absence of NEAT1 disrupts the metabolic transition to glycolysis in neutrophils, which in turn diminishes their pro-inflammatory activities. Our findings extend the existing knowledge of NEAT1 in UC pathogenesis, uncovering a novel target and molecular mechanism through which NEAT1 modulates intestinal inflammation. However, a whole-body NEAT1 knockout model used in our current study cannot completely exclude the potential contributions of other immune cell types. Future studies using neutrophil-specific NEAT1 knockout model will be required to definitively confirm the neutrophil-intrinsic role of NEAT1 in UC intetinal inflammation.

## Data Availability

The original contributions presented in the study are publicly available. This data can be found here: NCBI SRA, accession number PRJNA1510622.
